# Community participation in primary care: willingness to participate, a web survey in the Netherlands

**DOI:** 10.1017/S1463423618000695

**Published:** 2018-09-27

**Authors:** Madelon Kroneman, Kim van Erp, Peter Groenewegen

**Affiliations:** 1 PhD, Senior Researcher, NIVEL (Netherlands Institute of Health Services Research), Utrecht, The Netherlands; 2 PhD, Researcher, NIVEL (Netherlands Institute of Health Services Research), Utrecht, The Netherlands; 3 Researcher, VU University, Amsterdam, The Netherlands; 4 Professor, Department of Human Geography, Utrecht University, Utrecht, The Netherlands

**Keywords:** community participation, primary care, the Netherlands, survey

## Abstract

**Aim:**

The aim of the study is to explore to what extent members of the community are willing to participate in the way their primary care practice is organized and which characteristics of people and community are associated with this willingness.

**Background:**

Community participation in primary care refers to involvement of community members in the organization, governance and policy making of primary care facilities. Due to demographic changes and changes in the role of patients and the community concerning health care, it becomes important to include the social environment of patients into healthcare. Community participation may help GPs to improving their practice and providing care according to the needs of the population. Interpreted this way, it may be an important contributor to quality of care.

**Methods:**

In 2016, a web questionnaire was send to 800 members of the Dutch Health Care Consumer Panel. The response rate was 34%. Willingness to participate was divided into perceived readiness, ability and time to participate. The data were analysed using frequency tables and linear regression analysis.

**Findings:**

Half of the participants were ready to give their opinion on primary care and one-third reported willingness to participate in decision making. Participants were less optimistic about their ability to participate and the time they have available for participation. Readiness and perceived ability were mainly determined by the importance that the respondents attributed to participation. Participants with previous experience in volunteering appeared more willing to spend time on participation.

**Conclusions:**

This study showed that half of the respondents are willing to participate, but they are less sure about their ability to do so and that finding time to participate is seen as problematic. Future research should focus on which characteristics influence willingness. This knowledge might help primary care facilities to recruit people more easily and successfully.

## Introduction

Community participation in primary care refers to involvement of community members in the organization of primary care facilities, their policy making and governance of the care they provide. Community participation can take a wide variety of forms. For instance, patients may give their opinion about opening times of the facility, waiting room design, waiting times and responsiveness of the care providers. Furthermore, patients may be involved in setting priorities or discussing self-management in chronic care provision (eg, for diabetes) or may participate in the organization of preventive activities together with the primary care facility. Community participation in primary care becomes increasingly important due to several developments. First, health is no longer considered the absence of diseases but should be more broadly defined as the ability of people to adapt and self manage their health status in the face of social, physical and emotional challenges (Huber *et al*., 2011). Primary healthcare may support its patients in these adaptation and self-management processes. In addition to involving individual patients in their own care, community members could be consulted for improving care programs for, for instance, chronically ill, or for providing advice on the organization or policy making of primary care practices as a whole.

Second, epidemiologic and demographic developments influence healthcare delivery. The ageing of the population and the related increasing number of patients with multi-morbidity (OECD, 2011; Gijsen, 2013) requires a patient-centred rather than disease-centred approach, since the combination of diseases may require a different approach than each disease separately. Furthermore, especially because many chronic diseases are associated with (an unhealthy) lifestyle, this requires primary care to focus more on preventive measures (Ursum *et al*., 2011). Lifestyle changes are often difficult to achieve because patients’ lifestyle is usually deeply embedded in their social surroundings. Therefore, primary care should be more aware of and attentive to the social surroundings, and could do so by getting the community involved in primary care.

Third, the organization of healthcare, especially in the Netherlands, is changing. Recently, the organization of long-term care has been decentralized, with the aim to keep people at home as long as possible. Furthermore, the emphasis in long-term care lies on self-reliance of patients with the help and support of their social environment. As a result, it becomes increasingly important to know what patients expect from primary care. Additionally, informal carers are increasingly becoming an addition to primary care. The expectations, needs and preferences of patients and informal carers provide a valuable perspective on how to improve the organization of primary care in order to deal with this relatively new situation.

Involving the community in primary care is difficult. It is a challenge to find people who are willing to participate and to keep them motivated (Mann, 1985; Petsoulas, 2015). Participants are often not representative for the total patient population and as a result, professionals may question the validity of the input of these participants (Martin, 2008; Pollard *et al*., 2014). Especially women, men in the age of 16–29 years and people from deprived areas and lower social classes tend to be underrepresented (Agass *et al*., 1991; Segall, 2003; Petsoulas *et al*., 2015; Freeman *et al*., 2016). Although the urge for community participation in primary care is increasing (in the United Kingdom it is even legally obliged for GPs; Agass *et al*., 1991; Pollard *et al*., 2014) research on the circumstances or characteristics that may influence community members’ willingness to participate is scarce. In the light of the urge for community participation in primary care and the problems in finding participants, we explored the willingness of people to participate in primary care.

## Research aim

The aim of the present research is to explore to what extent people are willing to participate in primary care and which characteristics of people and the community are associated with this willingness.

First, we expect that the more importance community members attribute to participation, the more willing they will be to actually do so. Community members who consider participation as important see an added value in participation, for instance, to make valuable changes to their local situation. This awareness may function as a motivator to contribute. Therefore, we expect importance attached to community participation to be positively associated with willingness to participate.

Hypothesis 1: Willingness to participate in primary care is positively associated with importance attached to community participation.

Second, we expect willingness to participate is associated with the degree to which people have a personal interest in primary care, since they may directly benefit from their efforts to improve primary care practices. We therefore expect elderly people, people with a chronic illness and informal carers to be more willing to participate, since they make use of primary care more often than young and healthy individuals.

Hypothesis 2: Willingness to participate in primary care is positively associated with personal interest in primary care.

We expect that people who already have experience in community participation or are already involved in volunteering are more willing to participate. First, such previous experience reveals a general interest in contributing to the community. Second, we expect that experienced individuals know how to overcome the threshold to participate. That is, they know from experience how to free up time for such activities and they know how to overcome possible uneasiness in expressing their opinions.

Hypothesis 3: Willingness to participate in primary care is positively associated with (previous) experience in community participation and being active as a volunteer.

Finally, individuals with more resources at their disposal to participate may be more willing to participate. For instance, Van Houwelingen *et al*. (2014) showed that people with a higher education and higher income are more often involved in community participation in general than individuals with a low educational background or lower income. We therefore expect levels of education and income to be positively related to community members’ willingness to participate. A social resource that may influence willingness to participate is the availability of a strong social network in the neighbourhood and the existence of norms and values that supports and endorses community participation. We expect that people who live in communities with a strong social cohesion are more willing to participate than those in weaker social cohesion areas.

Hypothesis 4: Willingness to participate in primary care is positively associated with resources such as educational level, income and social cohesion.

Another incentive to participate may come from the threat that primary care services may disappear in one’s community. In areas where population is declining, the continued presence of nearby primary care services is often seriously threatened (Neuwelt, 2012). Therefore, we expect community members living in areas were the population is declining or is expected to decline to be more willing to participate in community participation projects than those who live in other areas.

Hypothesis 5: People living in areas were the population is declining are more willing to participate in primary care than participants living in non-declining areas.

In this study we focus on willingness to participate by citizens, but community participation also requires efforts from the primary care providers, such as GPs (‘it takes two to tango’). In another part of our research project (not published here), we found that in the Netherlands there are already promising participation projects going in. Of course there are GPs that are reluctant, but we found also several enthusiastic GPs who already implemented a divers selection of community participation(Groenewegen *et al*., 2016).

The influence of gender on willingness to participate is difficult to establish. In the literature we did not find clear explanations as to why women and young men appear to be less willing to participate. We therefore did not include a hypothesis on the effect of gender, but we included gender in the analyses as a control variable.

## Methods

### Participants

As part of a larger study on community participation a questionnaire was send to 800 members of NIVEL’s Dutch Health Care Consumer Panel. The Dutch Health Care Consumer Panel is an access panel consisting of about 12 000 members, from which samples can be drawn. The panel members share their expectations and experiences on health care via questionnaires (Brabers *et al*., 2015).

Questionnaires were sent by email – participants could use a hyperlink to get to the online questionnaire. The questionnaire was accessible from July to August 2016. Two reminders were sent to the participants. A total of 271 respondents returned questionnaires (overall response rate=34%). Twelve questionnaires were excluded because of incompleteness.

### Measures

#### Willingness to participate

Participants were provided with a fictitious example to participate in their GP practice. In this example, they were requested to participate, with ten other patients, in a meeting with their GP to discuss issues considered important to their neighbourhood (for the full questionnaire, see Supplement 1). Participants then answered five questions developed by the authors that measured their willingness to participate in this primary care project. We distinguished three dimensions of willingness to participate: (1) readiness to participate, measured by two items (‘I would like to give my opinion on the primary care practice of my GP/on my GP’s practice’ and ‘I would like to co-decide on the care and the supply of care within the practice’); (2) ability to participate, measured by two items (‘I have enough experience to participate in such a meeting’ and ‘I have sufficient experience to give my opinion in such a meeting’). Originally, we planned to include the question ‘I lack the knowledge on health care to contribute meaningfully to plans for improvement’, but we dropped this question, because it did not fit in the scale with the other two questions. Cronbach’s *α* dropped from 0.78 to 0.61; and (3) Available time, measured by one item (‘I am willing to spend time on such a meeting’). Questions were answered on a seven-point scale (1=completely disagree, 7=completely agree).

The three dimensions of willingness are to some extend related, but certainly not the same as becomes clear from the correlation matrix (Table 1).

**Table 1:** Pairwise Pearson correlation coefficients of the three dimensions of willingness to participate

Dimensions of willingness	Readiness Correlation coefficient Significance Number of observation	Ability Correlation coefficient Significance Number of observation	Time Correlation coefficient Significance Number of observation
Readiness			
Correlation coefficient	1.000		
Significance			
Number of observation	*N*=258		
Ability			
Correlation coefficient	0.716	1.000	
Significance	0.000		
Number of observation	*N*=256	*N*=257	
Time			
Correlation coefficient	0.596	0.699	1.000
Significance	0.000	0.000	
Number of observation	*N*=256	*N*=256	*N*=257

#### Importance given to participation

Importance addressed to participation was measured with four questions. Patients indicated the importance of (1) getting informed about the primary care organization, (2) having the option to give their opinion on the way primary care is organized, (3) sharing in decision making on the organization of the practice and (4) care providers informing patients on the result of their participation. All items were measured on a seven-point scale. The four items together had a Cronbach’s *α* of 0.89 and the principal component analyses revealed that 75% of variance was explained by one factor that combines the four items. Therefore, the variables were combined in one measure of importance by calculating the mean score for each respondent. For Table 3, the variable was divided into three categories (score 1–5: not (so) important; 5.25–6: important; 6.25–7: very important).

#### Personal interest in primary care: age, informal care provision, chronic disease presence

Age was measured in years. We asked participants to indicate whether they provided *informal care* for a family member or relative, and if so, how many hours a week. We replaced values for hours of informal care for those individuals that indicate to spend on average more than 8 hours a day, 7 days per week on informal care, because this seems physically unlikely. The values were replaced with the next highest reported hours spend on informal care, which was 46 hours. The presence of specific chronic diseases (35 conditions) is known for panel members. We selected those conditions that are treatable in primary care. For some conditions, this is obvious (eg, hay fever), and when we were not sure, we made use of the list of ambulatory care sensitive conditions for which hospitalization can be prevented when treated in primary care, published by Sundmacher *et al*. (2015). We distinguished between participants with none (=0), one or two (=1) and three or more (=2) primary care treatable chronic conditions (see Table S1).

#### Having experience: active community members

Active community members where those who reported to have previously been active in community participation (both in general and in primary care) or to be active in volunteering.

#### Resources to participate: education, income and social cohesion

We distinguished three categories in educational level (1=lower, 2= middle and 3= higher education). The variable nett income is coded into four categories of equal size (1=less than 1900 euros per month, 2= between 1900 and 2499 euro per month, 3=between 2500 and 3500 per month, 4=more than 3500 per month). Using the four digits of the postal code of the participant’s home address we linked data on social cohesion to that postal code area. The measure of social cohesion was aggregated from a large Dutch population survey. It indicates the degree to which the cohesion of the area where people live differs from the grand mean in the Netherlands in general (Waverijn *et al*., 2017). Social cohesion was coded into three groups of equal size (see Table S2).

#### Declining areas

Again using the four digit postal code we generated a variable that indicated whether (= 1) or not (=0) participants lived in areas that cope with population decline, now or in the near future. Data on declining areas came from Statistics Netherlands.

#### Control variables

Gender (1=male, 2=female) was used as a control variable.

### Data analyses

The statistical analysis was performed by ordinary linear regression using STATA 14.

### Research ethics

Participation in the Dutch Health Care Consumer Panel is on a voluntary basis. People give their informed consent at the start of their participation in the panel. No further research ethics approval is needed according to Dutch data protection legislation. The Panel is registered with the Dutch Data Protection Authority under number 1262949.

## Results

When comparing our sample with the general population, we found that our sample has relatively more middle aged and elderly persons compared to the Dutch population and our sample is higher educated (see Table 2).

**Table 2:** The research population compared with the general Dutch population

	Research sample (*n*=258)		Dutch population^a^
Gender	*N*	%	%
Male	137	53	49
Female	121	47	51
Age			
18–39 years	56	22	34
40–64 years	126	49	45
65–79 years	65	25	16
80 years and older	11	4	5
Education			
Low	26	10	30
Middle	113	44	42
High	116	45	28

a Statistics Netherlands, 2014.

**Figure 1:**
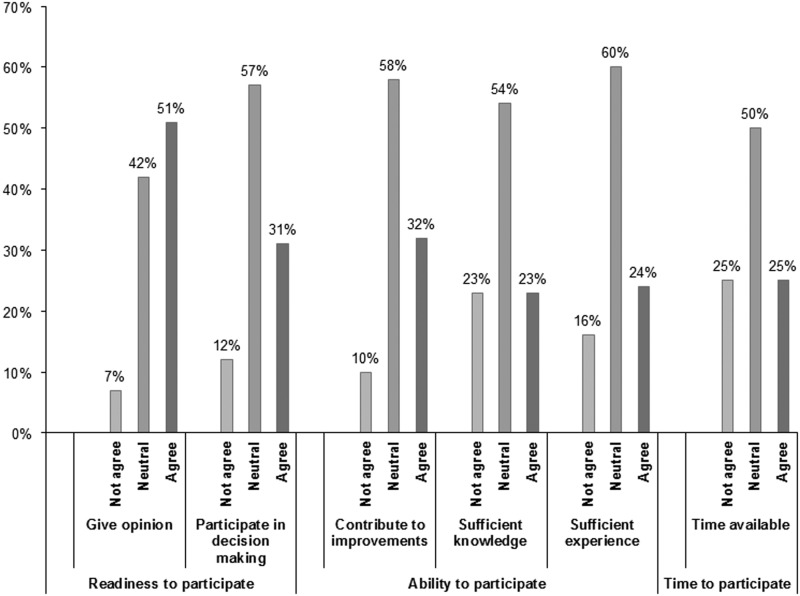
Readiness, ability and time to participate as reported by the respondents.

When looking at the individual items that describe the willingness to participate, we found that half of the participants prefer giving their opinion on their GP practice and about one third would also prefer to participate in decision making concerning the organization of care of their general practice. About their ability to participate, participants are slightly less optimistic. About one-third thinks they can contribute meaningfully, but only a quarter thinks they have enough knowledge and experience. Only a quarter of the participants think they have time available to participate (see Figure 1).

**Figure 2:**
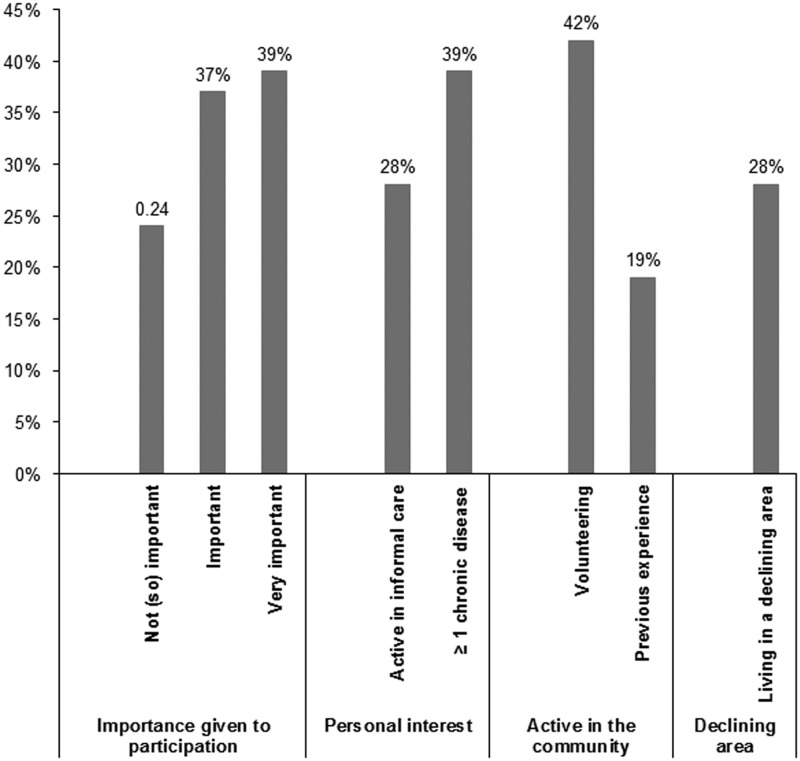
Distribution of the independent variables as described in the hypotheses.

The participants overall find it important to participate in primary care. Three out of every ten participants provide informal care for family members or relatives and four out of ten have at least one chronic condition (Figure 2). People have more experience in volunteering (42%) compared to community participation projects (19%). Our data on volunteering are in line with the data of the Netherlands Institute for Social Research, that finds similar percentages of people performing voluntary work (Dekker and De Hart, 2009).

Readiness, ability, and time to participate in primary care depend on the importance people attached to primary care participation (Table 3). Only when they were asked whether they have time to participate, other characteristics play a role. People who have previous experience with community participation tend to be more positive about spending time on primary care participation. People with an income in the third quartile are more willing to spend time compared to the other income categories. Surprisingly, people who live in a declining area appear to be less willing to spend time on patient participation. Social cohesion does not appear to be a resource that facilitates participation in the organization of primary care. Formally not significant, but very close to it, highly educated people considered themselves more able to participate than lower educated people (*P*=0.055). Furthermore, people living in an area with a high social cohesion appear to be less willing to spend time on participation (*P*=0.053). The latter is in contrast to our expectations.

**Table 3:** Linear regression analyses on readiness, ability and time to participate in the organization of primary care

	Readiness		Ability		Time	
	B	SE	B	SE	B	SE
Importance	**0**.**78**	0.07**	**0.66**	0.07**	**0.59**	0.09**
Personal interest						
Hours spend on informal care	0.01	0.01	0.01	0.01	−0.00	0.02
Chronic conditions treatable in primary care (none=reference)						
1–2 conditions	−0.17	0.19	−0.13	0.19	−0.10	0.24
3 or more conditions	0.19	0.28	0.52	0.28	0.05	0.36
Age	0.00	0.01	−0.01	0.01	0.01	0.01
Active in the community						
Experience (no=reference)	0.10	0.22	0.45	0.23	**0.78**	0.29**
Hours volunteering	0.01	0.02	−0.01	0.02	0.03	0.03
Resources						
Education (low=reference)						
Middle	0.21	0.30	0.46	0.30	0.50	0.39
High	−0.03	0.32	0.62	0.32^–^	0.51	0.41
Income category (low=reference)						
Second quartile	−0.20	0.23	−0.03	0.23	0.35	0.30
Third quartile	0.09	0.24	0.15	0.24	**0.69**	0.31*
Fourth quartile	0.08	0.26	0.11	0.26	0.48	0.33
Cohesion of neighbourhood	−0.39	0.83	−0.19	0.83	−2.07	1.06^–^
Living in a declining area (no=reference)	−0.03	0.19	−0.32	0.19	**−0.71**	0.24**
Control variable						
Sex (male=reference)	−0.18	0.18	−0.04	0.18	0.30	0.23
Intercept	−0.25		−0.29		−1.38	
Number of observations	208		209		210	
Adjusted *R* ^2^	0.37		0.30		0.24	

*P<0.05, **P<0.01, ^–^ *P*<0.06.

## Discussion

In this study, we distinguished three types of willingness to participate: readiness to participate, which indicates whether people are in principle willing to participate; ability to participate, indicating whether people consider themselves able to participate; and time to participate: do people think they can find time for participation activities. In line with our expectations, the more importance participants attached to community participation the more willing they were to participate. This positive association emerged for all three aspects of willingness: readiness, ability and time (Hypothesis 1). We expected that people who have a personal interest to be involved in primary care (Hypothesis 2) would be more willing to participate, but this was not the case. Since primary care is the first healthcare provider people in the Netherlands will contact in the case of health complaints, almost all Dutch citizens visit their primary care provider once in a while. Possibly, every patient may think they have enough of an interest in primary care, not only those who may have more regular contact as a result of their condition or as a result of taking care of a sick person. People who previously participated in community activities appear to be more willing to spend time on participation in primary care (Hypothesis 3). This positive association did not emerge for readiness and ability to participate. This may indicate that once people are persuaded to participate, they may be more willing to continue to do so when asked in the future. Personal resources, such as income, education and environmental factors as social cohesion and living in a declining area hardly show the hypothesized association with willingness (Hypothesis 4). Only respondents with an income in the third quartile appeared more willing to spend time on community participation in primary care. Living in a declining area had a negative association with the willingness to spend time on community participation, which was contrary to our expectation (Hypothesis 5). At present, we do not have an explanation for this finding; however, it might be related to distance to a GP practice, which is larger in areas with population decline (mostly rural areas).

In the literature, several problems with patient participation are mentioned. The most important from the side of patients are the lack of interest of citizens and the fact that the participants do not form a representative share of the population (Agass *et al*., 1991; Segall, 2003; Martin, 2008; Pollard *et al*., 2014; Petsoulas *et al*., 2015; Freeman *et al*., 2016). Our study revealed that most people find patient participation important (about three quarter of our respondents) and that readiness and ability to participate are not related to age, gender, income, education or personal interest in primary care. Only when people are asked to spend time, one of the expected differences in willingness emerged: previous experience as a volunteer seems to lower the threshold to participate.

A strong point of our study is the division of willingness in three different aspects: readiness, ability and time. In a previous study (Groenewegen *et al*., 2016), we found that this difference is essential: people may be ready to participate, feel able to participate, but having (no) time to participate may still hamper the actual participation.

A weak point is the relatively low response rate, which may be due to the fact that the questionnaire was send in the summer season and many respondents may have been on holiday. Unfortunately, this was dictated by the planning of the broader project of which the survey was part. Using the Dutch Healthcare Consumer Panel may have introduced bias towards people who are willing to participate. After all, the fact that these people are willing to be part of an access panel and to fill out questionnaires on healthcare related issues is in itself an indication of participation.

Community participation in primary care is still in its infancy in the Netherlands. There is no obligation for primary care practices to involve members of the community. Traditionally, only a number of integrated health centres have or had a patient board. However, with changes in epidemiology and healthcare policy, the interest of primary care practices to engage the community might be on the increase.

## Conclusion

This study showed that half of the respondents are willing to participate, but that they are less sure about their ability to do so and that finding time to participate is seen as problematic. There is no (clear) influence of age, income, education and personal interest on willingness to participate. Since these ‘traditional’ indicators do not relate to willingness, we suggest that future research should focus on which characteristics do influence willingness. Knowledge about these characteristics might help primary care facilities to recruit people. It is no problem if these characteristics are not the standard socio-demographic characteristics. GPs know their patients personally, seeing most of them more or less regularly in the consultation room. Knowing which characteristics are important for predicting willingness to participate might help primary care facilities to recruit people more easily and successfully.

## Financial support

This work was supported by a grant from Versterking Eerste Lijn Zuid Nederland

## Conflicts of interest

None.

## Supplementary material

For supplementary material accompanying this paper visit https://doi.org/10.1017/S1463423618000695. click here to view supplementary material
