# Supplementary material for: Community participation in primary care: willingness to participate, a web survey in the Netherlands
Source: Prim Health Care Res Dev. 2018 Sep 27;20:e13. doi: 10.1017/S1463423618000695 (PMC6476338; doi:10.1017/S1463423618000695)
Supplement: Supplementary file 1 [file S1463423618000695sup.zip › S1463423618000695sup001.docx]

**Table S1. Readiness, ability and time to participate of the respondents**

|  |  |  | **n** | **%** |
| --- | --- | --- | --- | --- |
| **Readiness to participate** | | |  |  |
|  | Prefer to give opinion on GP practice | |  |  |
|  |  | (Completely) not agree | 18 | 7 |
|  |  | Neutral | 108 | 42 |
|  |  | (Completely) agree | 132 | 51 |
|  | Prefer to participate in decision making | | |  |
|  |  | (Completely) not agree | 31 | 12 |
|  |  | Neutral | 148 | 57 |
|  |  | (Completely) agree | 80 | 31 |
| **Ability to participate** | | |  |  |
|  | Can contribute meaningfully to improvements | | |  |
|  |  | (Completely) not agree | 25 | 10 |
|  |  | Neutral | 150 | 58 |
|  |  | (Completely) agree | 82 | 32 |
|  | Have sufficient knowledge | |  |  |
|  |  | (Completely) not agree | 60 | 23 |
|  |  | Neutral | 140 | 54 |
|  |  | (Completely) agree | 59 | 23 |
|  | Have sufficient experience | |  |  |
|  |  | (Completely) not agree | 41 | 16 |
|  |  | Neutral | 155 | 60 |
|  |  | (Completely) agree | 62 | 24 |
| **Time to participate** | | |  |  |
|  | Have time available for participation activities | | |  |
|  |  | (Completely) not agree | 64 | 25 |
|  |  | Neutral | 128 | 50 |
|  |  | (Completely) agree | 65 | 25 |
|  |  |  |  |  |
|  |  |  |  |  |
| Items on readiness, ability and time to participate are measured on a 7-point scale. The values 1 and 2 are combined in "(Completely) not agree". Values 3 to 5 into "Neutral" and 6-7 into "(Completely) agree". Have sufficient knowledge was recoded to obtain the same direction. Have sufficient knowledge was not included in the analyses of willingness to participate, because it did not fit in the scale of ability to participate. | | | | |
