# Supplementary material for: Community participation in primary care: willingness to participate, a web survey in the Netherlands
Source: Prim Health Care Res Dev. 2018 Sep 27;20:e13. doi: 10.1017/S1463423618000695 (PMC6476338; doi:10.1017/S1463423618000695)
Supplement: Supplementary file 1 [file S1463423618000695sup.zip › S1463423618000695sup002.docx]

**Table S2. Frequency distribution of the independent variables from the hypotheses**

|  |  |  | **n** | **%** |
| --- | --- | --- | --- | --- |
| **Importance given to participation** | | |  |  |
|  |  | Not (so) important | 61 | 24 |
|  |  | Important | 97 | 37 |
|  |  | Very important | 101 | 39 |
| **Personal interest** | | |  |  |
|  | Respondents active in informal care | | 71 | 28 |
|  | One or more chronic disease | | 102 | 39 |
| **Active in the community** | | |  |  |
|  | Volunteering | | 107 | 42 |
|  | Previous experience in comm. part. | | 49 | 19 |
| **Resources** | | |  |  |
|  | Social cohesion of neighbourhood^1)^ | |  |  |
|  |  | Weak | 77 | 33 |
|  |  | Intermediate | 72 | 31 |
|  |  | Strong | 85 | 36 |
|  | Income^2)^ | |  |  |
|  |  | Up to €1,900 net per month | 69 | 28 |
|  |  | €1,900 - €2,500 net per month | 56 | 23 |
|  |  | €2,500 - €3,500 net per month | 64 | 26 |
|  |  | More than €3,500 | 58 | 23 |
| **Declining area** | |  |  |  |
|  | Living in a (future) declining area | | 73 | 28 |
| ^1)^Cohesion is divided into three equal groups | | |  |  |
| ^2)^Income is divided into four equal groups | | |  |  |
